# Anesthetic Considerations for Elective Laparoscopic Cholecystectomy in a Patient With Previous Pneumonectomy

**DOI:** 10.7759/cureus.22176

**Published:** 2022-02-13

**Authors:** Sharad Kumar, Vamsi Krishna Uppalapati, Rajiv Shukla, Ashok Chattoraj

**Affiliations:** 1 Anaesthesiology, Tata Main Hospital, Jamshedpur, IND; 2 General Surgery, Tata Main Hospital, Jamshedpur, IND

**Keywords:** spirometry, pulmonary function test, anesthesia, laparoscopic cholecystectomy, post pneumonectomy

## Abstract

Patients presenting for surgery after pneumonectomy pose significant challenges to anesthesiologists. The disease process necessitating pneumonectomy may involve the surviving lung too. Cholecystectomy is a major surgery, and the open approach has significant risks of post-operative pulmonary complications in these patients partly owing to the large incision and postoperative atelectasis, associated with inadequate post-operative analgesia. Contemplating a laparoscopic procedure in patients with a single, possibly damaged lung, involves a good understanding of the physiology of the single lung as well as the challenges posed by capnoperitoneum. Here, we present a case of a female with a history of previous pneumonectomy undergoing laparoscopic cholecystectomy. There are very few reports of patients after pneumonectomy who have subsequently undergone a laparoscopic cholecystectomy successfully and this report highlights some crucial factors to be kept in mind during anesthetic management of such patients.

## Introduction

Patients with pulmonary diseases are high risk for general anesthesia due to increased risk of perioperative respiratory complications. Pneumonectomy is an example of extreme pulmonary compromise induced by a therapeutic surgical resection [1,2]. Early and late post-pneumonectomy morbidity can be high, ranging between 40%-60%. Any further elective or emergency surgical procedure in these patients adds to the existing concerns. Post-pneumonectomy anatomical and physiological changes are predictable and a good understanding of this is mandatory for the successful management of such patients [3,4].

Most published data about surgical and anesthetic experience in patients with a history of pneumonectomy involves cardio-pulmonary procedures, but data on anesthesia concerns during laparoscopic cholecystectomy in patients with pneumonectomy is scarce. A careful pre-anesthetic pulmonary function evaluation and optimization is the essence of management of such cases. In this case report, we describe the management of one such patient who underwent laparoscopic cholecystectomy, eleven years after left pneumonectomy.

## Case presentation

A 55-year-old, 68 kilogram (kg) female was scheduled for cholecystectomy in our hospital for symptomatic gallstone disease. The patient had undergone left pneumonectomy eleven years back for multiple abscesses of the lung from pulmonary tuberculosis. The right lung had also been affected, however, no segmental resection was required then, as per records available with the patient. The patient had received a full course of anti-tubercular treatment. With physiotherapy and exercise training, she had regained good effort tolerance and was able to climb two flights of stairs and complete all routine work without breathlessness. After discussions with the operating team, a decision to take the laparoscopic approach was made and a thorough preoperative evaluation was done. The patient had a history of hypothyroidism which was controlled on tablet thyroxine 50 microgram (mcg) every day; she also had hypertension for which she was not taking any medications.

**Figure 1:**
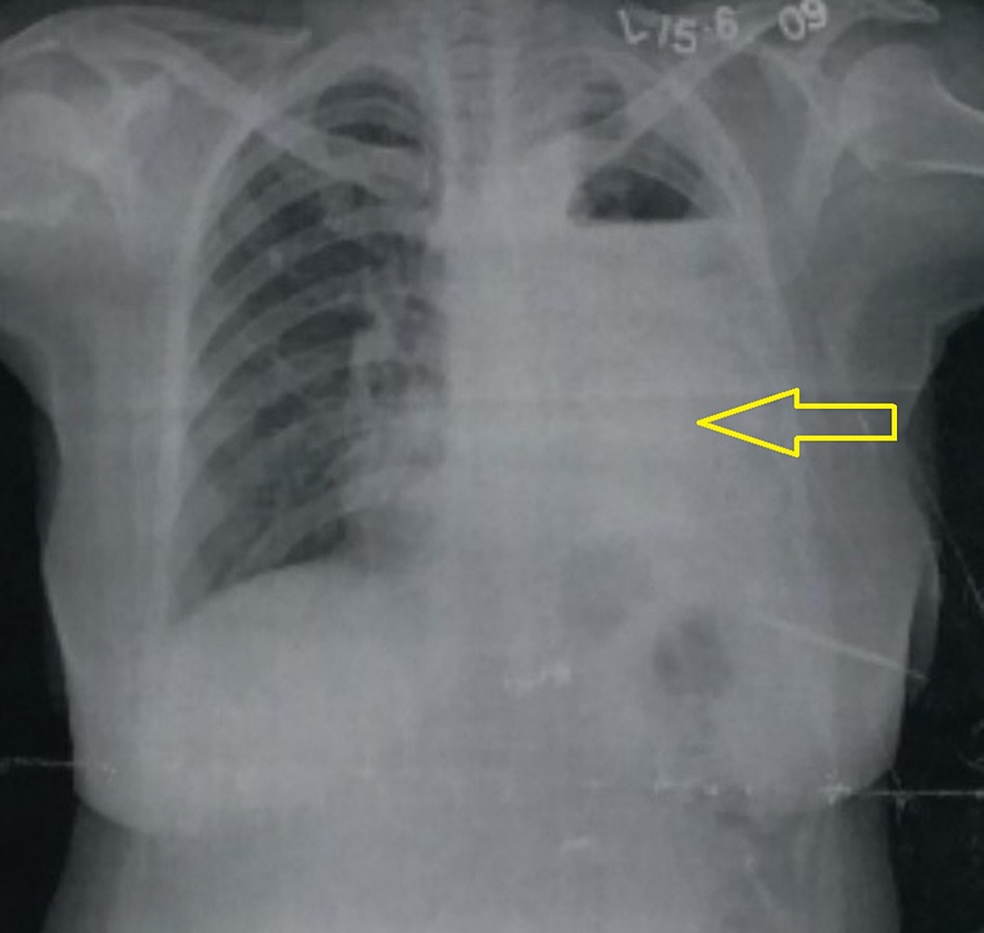
Chest X-ray demonstrating left pneumonectomy status with right functional lung.

On examination, a post-operative surgical scar of 8 centimeter (cm) in the left chest wall was identified. She was hemodynamically stable, and her respiratory rate was 20 breaths per minute with regular rhythm and adequate depth. Her oxygen saturation was 95% on room air. The clinical examination indicated a leftward shift of the apical impulse and radiological features confirmed a left mediastinal shift as expected in such patients. Air entry was absent throughout the left chest and was noted to be diminished on the right side too. Heart sounds were muffled but no obvious added sounds or murmurs could be auscultated. Her chest X-ray showed left pneumonectomy status with right functional lung, which did not show any radiological evidence of residual tuberculosis (Figure 1).

**Figure 2:**
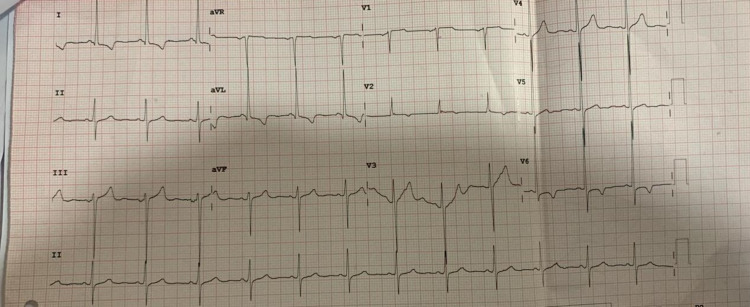
Electrocardiogram showing left ventricular hypertrophy, but no signs of right ventricular hypertrophy or for pulmonale.

**Figure 3:**
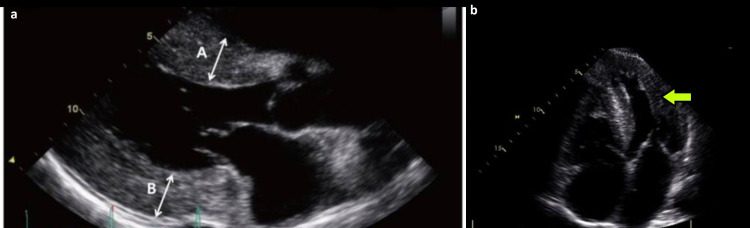
Echocardiography showing left ventricular hypertrophy - parasternal long-axis (a) and apical four chamber (b) view. A: interventricular septum; B: left ventricle posterior wall

Electrocardiography (ECG) showed left ventricular hypertrophy (LVH) pattern and echocardiogram confirmed no structural abnormalities except for the mild LVH. There was no evidence of right ventricular hypertrophy or for pulmonale on her ECG or echo (Figures 2-3).

Her breath-holding time was 25 seconds. Spirometry revealed values as shown in Table 1.

**Table 1:** Spirometry result on admission. FVC: forced vital capacity; FEV1: forced expiratory volume in one second; FEF 25%-75%: mean forced expiratory flow during the middle of FVC; FEF 75%-85%: forced end-expiratory flow; PEF: peak expiratory flow; FIVC: forced inspiratory vital capacity; FIV1: forced inspiratory volume in one second; PIF: peak inspiratory flow

Parameter	Measured	Predicted	% Predicted
FVC (L)	0.82	2.28	36
FEV1 (L)	0.81	1.92	42
FEV1/FVC	98.3	78.5	125
FEF 25-75% (L/sec)	1.29	2.92	44
FEF 75-85% (L/sec)	0.54	-	-
PEF (L/sec)	2.85	5.44	52
FEF 25% (L/sec)	2.81	5.02	56
FEF 50% (L/sec)	1.53	3.43	45
FEF 75% (L/sec)	0.59	1.3	46
FIVC (L)	0.76	-	-
FIV1 (L)	0.75	-	-
FIV1/FIVC	99.3	-	-
PIF (L/sec)	0.83	-	-

Spirometry results revealed a restrictive lung pattern. Both forced vital capacity (FVC) and forced expiratory volume in one second (FEV1) were reduced at 36% and 42% of predicted values, respectively. However, an FEV1 value of 0.81 liters (L) encouraged us to go ahead with the laparoscopic approach. A preoperative arterial blood gas analysis revealed pH of 7.40, partial pressure of oxygen (PO_2_) of 81 mmHg, partial pressure of carbon dioxide (PCO_2_) of 34.6, bicarbonate ion (HCO3^-^) of 30 mmol/L, and base deficit of -1.6 mmol/L.

After taking a high risk-informed consent in view of the history of pneumonectomy and severely restrictive pulmonary pattern, the patient was taken up for surgery under general anesthesia. Monitors for ECG, non-invasive blood pressure (NIBP), oxygen saturation, and temperature monitoring were attached. Invasive arterial line was secured under local anesthesia to monitor blood gas as well as invasive blood pressure.

General anesthesia was induced by administering intravenous midazolam 1 milligram (mg), injection dexamethasone 6 mg, injection fentanyl 150 mcg, followed by injection propofol 100 mg and injection atracurium 35 mg. Airway was secured using a 7 millimeter (mm) cuffed endotracheal tube fixed at 18 cm confirming adequate right-sided air entry. General anesthesia was maintained with oxygen in air and sevoflurane at 1-1.5 minimum alveolar concentration (MAC). Surgeons were requested to create pneumoperitoneum at a slow rate and to keep the intra-abdominal pressures as low as possible. At no time did the intraperitoneal pressure rise beyond 12 mmHg during surgery. Pressure control mode was used for ventilation to achieve the target of expired tidal volume of 300-350 ml keeping peak pressures below 30 mmHg. Respiratory rate was maintained to achieve end-tidal carbon dioxide (ETCO_2_) pressure between 35 and 40 mmHg. With pneumoperitoneum, the airway pressures also increased significantly. This was partly offset by increasing the inspiratory time to keep the inspiratory to expiratory phase ratio at 1:1. Positive end-expiratory pressure (PEEP) of 5 cm of water pressure was kept. Intraoperatively, one liter of crystalloids was infused. Hemodynamic parameters remained stable intraoperatively. The patient was extubated successfully by reversing neuromuscular blockade with 2.5 mg neostigmine and 0.5 mg glycopyrrolate. Intravenous paracetamol 1 gram (gm) and injection ondansetron 4 mg was administered before extubating. The patient was observed in a high-dependency unit overnight, transferred to the general ward the next day, and discharged on the second postoperative day in a stable condition.

## Discussion

The post pneumonectomy state is associated with a severely diminished respiratory reserve and predictable anatomic and physiological changes such as deviation of the heart and the mediastinum toward the side of the resected lung, compensatory hyperinflation, and herniation across the midline of the remaining lung. The mediastinum may rotate toward the vacant pleural space, causing extrinsic airway and esophageal compression. Mild thoracic scoliosis is also commonly encountered secondary to the changes in the shape of the thoracic cage. Postpneumonectomy syndrome is a rare syndrome of dynamic airway obstruction caused by extreme rotation and shift of the mediastinum after pneumonectomy, resulting in symptomatic central airway compression. There are reports of this syndrome, being managed in the extreme state, by mediastinal repositioning and placement of saline-filled prostheses into the pneumonectomy space [5].

There are several factors which influence the pulmonary function in patients after pneumonectomy such as the preoperative function of the remaining lung, the patient’s age, the extent of postoperative compensatory changes, the duration of time elapsed after pneumonectomy, and site of lung resected [6,7]. Right pneumonectomy is associated with a threefold greater mortality than left pneumonectomy.

Capnoperitoneum created during laparoscopic procedures results in significant hemodynamic, as well as respiratory compromise. Allowing a laparoscopic surgery in post-pneumonectomy patients involves balancing the benefits and risks against that of an open surgery. A good preoperative evaluation must focus on assessing whether the patient will be able to tolerate these changes intraoperatively.

In our patient, the pre-operative lung function was not severely compromised. An FEV1 value of 0.8 L permitted us to contemplate laparoscopic surgery. There are centers that allow lobectomy procedures on patients based on either a predicted postoperative FVC of >800 ml/m^2^ or an FEV1 of >600 ml/m^2 ^[8]. They report zero percent mortality which indicates that this criteria can be accepted. Furthermore, eleven years had elapsed post pneumonectomy for our patient allowing enough time for all compensatory adjustments to have been made and internal homeostasis to have been achieved. Her ECG and echo showed significant left ventricular hypertrophy probably as a result of poorly controlled hypertension but there was no sign of right ventricular hypertrophy or pulmonale.

We could find only three existing reports on anesthesia approach for managing laparoscopic surgery in patients after pneumonectomy. Nair et al. and Newington et al. managed laparoscopic surgery after pneumonectomy using endotracheal tube and general anesthesia with mechanical ventilation (pressure-controlled ventilation), keeping the tidal volume (TV) of 5-6 ml/kg producing a peak inspiratory pressure (PIP) of 25 cm H_2_O [1,9]. We had also targeted a similar tidal volume and had fixed the pressure releasing valve at 30 mmHg to prevent airway pressures from ever crossing this value. Increasing the inspiratory time allowed us to increase the tidal volumes and minute ventilation without breaching the peak pressures set by us. Upon release of pneumoperitoneum, we applied extrinsic PEEP and employed recruitment maneuvers to minimize alveolar dead space and post-operative atelectasis. It was also beneficial to the patient that the cholecystectomy could be performed laparoscopically because it avoided post-operative pain and respiratory depression.

As the five-year survival rate of post pneumonectomy patients ranges from 40% to 75% [9,10], anesthesiologists should be prepared to encounter such patients for elective and emergency procedures. Understanding the physiological changes of pneumoperitoneum in a post pneumonectomy patient is the key to successfully managing such patients intraoperatively. Care during intraoperative positioning, minimizing the hemodynamic fluctuations, optimizing the fluid balance, and adequate analgesia are critical to achieving a smooth anesthesia experience. The main basic goal in the management of these cases remains to avoid hypoxemia and hypercarbia.

## Conclusions

Laparoscopic cholecystectomy is feasible and may be safely considered for post-pneumonectomy patients. Laparoscopic procedures also have the advantage of reduced postoperative pain and requirement for opioid medications which have a significant respiratory depressant effect. A complete understanding of the physiological changes of both pneumoperitoneum and post pneumonectomy status with a careful pre-operative evaluation of the clinical and pulmonary function is crucial in managing these patients. Efforts should be made to preserve the function of the remaining lung using a lung-protective ventilation strategy along with strict hemodynamic monitoring. Anesthesiologists must also be aware of rare and catastrophic complications like post-pneumonectomy syndrome, and pneumomediastinum should always be created slowly and carefully. It is always beneficial to keep the intra-abdominal pressures low in such patients. More published reports are required to guide successful anesthetic management of post-pneumonectomy patients during laparoscopic procedures other than cholecystectomy.
